# Six-year prognosis of anxiety and depression caseness and their comorbidity in a prospective population-based adult sample

**DOI:** 10.1186/s12889-022-13966-4

**Published:** 2022-08-15

**Authors:** Olivia Stålner, Steven Nordin, Guy Madison

**Affiliations:** Department of Psychology, Umeå University, 90187 Umeå, Sweden

**Keywords:** Affective disorder, Epidemiology, Incidence, Longitudinal, Association

## Abstract

**Background:**

Anxiety and depression are amongst the most prevalent mental health problems. Their pattern of comorbidity may inform about their etiology and effective treatment, but such research is sparse. Here, we document long-term prognosis of affective caseness (high probability of being a clinical case) of anxiety and depression, their comorbidity, and a no-caseness condition at three time-points across six years, and identify the most common prognoses of these four conditions.

**Methods:**

Longitudinal population-based data were collected from 1,837 participants in 2010, 2013 and 2016. Based on the Hospital Anxiety and Depression Scale they formed the four groups of anxiety, depression and comorbidity caseness, and no caseness at baseline.

**Results:**

The three-year associations show that it was most common to recover when being an anxiety, depression or comorbidity caseness (36.8 − 59.4%), and when not being a caseness to remain so (89.2%). It was also rather common to remain in the same caseness condition after three years (18.7 − 39.1%). In comorbidity it was more likely to recover from depression (21.1%) than from anxiety (5.4%), and being no caseness it was more likely to develop anxiety (5.9%) than depression (1.7%). The most common six-year prognoses were recovering from the affective caseness conditions at 3-year follow-up (YFU), and remain recovered at 6-YFU, and as no caseness to remain so across the six years. The second most common prognoses in the affective conditions were to remain as caseness at both 3-YFU and 6-YFU, and in no caseness to remain so at 3-YFU, but develop anxiety at 6-YFU.

**Conclusions:**

The results suggest that only 37 − 60% of individuals in the general population with high probability of being a clinical case with anxiety, depression, and their comorbidity will recover within a three-year period, and that it is rather common to remain with these affective conditions after 6 years. These poor prognoses, for comorbidity in particular, highlight the need for intensified alertness of their prevalence and enabling treatment in the general population.

## Background

Globally, more than 264 million people suffer from depressive disorders, and 284 million from anxiety disorders [1]. The estimated lifetime risk of developing an anxiety and depressive disorder is 9–18% and 12–25%, respectively [2, 3]. Examples of point prevalent rates for specific types of anxiety and depression in Sweden are 8.8% for generalized anxiety disorder, and 5.2% for major depressive disorder. These conditions are associated with poor quality of life [4], and rank very high among diseases regarding years lived with disability [5]. However, both anxiety and depression are undertreated conditions, with men and the elderly being especially reluctant to seek treatment [4, 6].

There is substantial comorbidity between anxiety and depression [4, 7, 8]. An overlap in prevalence, symptoms and risk factors [9] have resulted in terms such as cothymia and anxious depression [7, 8, 10, 11]. Compared to depression alone, comorbidity with anxiety may result in poorer recovery and treatment outcome [8, 12, 13], increased risk for suicide [3], and greater symptom severity [4, 7, 8, 14]. Individuals with comorbidity also report higher rates of somatic symptoms and lower quality of life compared to those with only one condition [4, 15]. Hence, early prevention of anxiety, depression, and, in particular, their comorbidity is important to decrease the risk of development of additional mental and somatic health problems.

A meta-analysis regarding both symptoms and disorder prevalence shows that anxiety and depression are bidirectional risk factors for one another [16], although anxiety appears to precede depression more commonly than the opposite direction [11]. However, their causal relationship remains uncharted territory [9, 16], in particular regarding long-term prognosis and potential change in condition in-between two endpoints in time. Understanding these changes over time in the general population is particularly important from a public health perspective, as a basis for the prevention of mental ill-health.

The purpose of the present study was to enhance the understanding for long-term prognosis of anxiety and depression caseness and their comorbidity in a general adult population. To this end we employed a population-representative dataset with self-reported health assessment at three time points, each time point separated by a three-year interval. Whereas it is documented that comorbidity is associated with worse severity and prognosis of anxiety and depression [17], the present design with a unique six-year span and three assessments was expected to provide further understanding for the role of comorbidity over a long time-period. Caseness refers to scores that meet a cut-off representing a high probability of being a clinical case [18], and was chosen as measure to enhance interpretation of the results, and increase its clinical relevance. This was conducted by following groups over time that at baseline either met criteria for anxiety caseness (but not depression caseness), depression caseness (but not anxiety caseness), comorbidity of anxiety and depression caseness, or none of these caseness criteria. A first objective was to investigate to what extent belonging to one of these caseness groups at baseline is associated with belonging to a certain caseness group three years later in a general adult population. For this purpose we took advantage of all available data, thus both from baseline to the 3-year follow-up (YFU) and from 3-YFU to 6-YFU. A second objective was to identify, among 64 possible prognoses, the most common prognoses across six years for each of the four conditions at baseline.

## Methods

### Population and sample

We used data from the Västerbotten Environmental Health Study [19], a population-based prospective cohort survey with a sample of residents in the county of Västerbotten in Northern Sweden. Västerbotten has an age and sex distribution that is very similar to that of the general Swedish population [20].

The sample size at baseline, in 2010, was based on the lowest expected prevalence for a specific environmental intolerance by sex (1.1%) [21]. Precision was set to 0.55% [22] and confidence level to 95%. The calculated sample size for men was 1382 by following a procedure of Daniel [23]. Since sex was almost equally distributed in Västerbotten (50.3% men) in 2010 [20], the number of women needed was set to the same number as for men. With an expected response rate of 60%, an expected accessibility of 90% and an expected response rate of 60% at 3-year follow-up (3-YFU), in 2013, the sample size was estimated to 8,530 participants at baseline, rounded up to 8,600. A sample was randomly selected by means of the municipal register after stratification for sex and the six age strata 18–29, 30–39, 40–49, 50–59, 60–69 and 70–79 years.

**Fig. 1:**
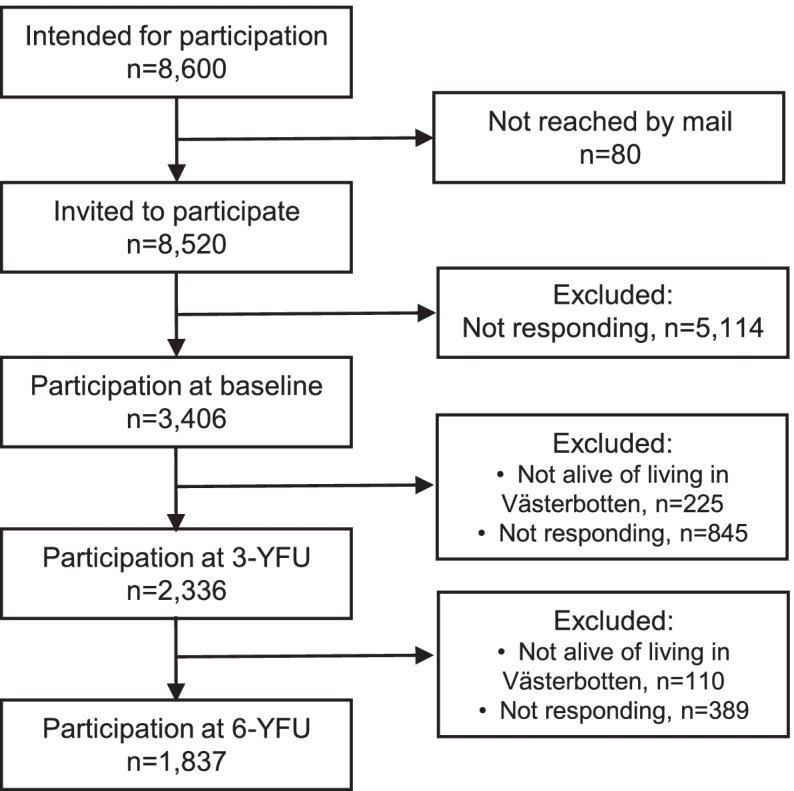
Flowchart depicting participation in the current study, including causes for exclusion of participants

A flowchart showing participation in the current study, including causes for exclusion of participants is given in Fig. 1. Eighty persons were excluded because they could not be reached by surface mail, leaving 8,520 persons. Of these, 3,406 (40.0%) responded to the questionnaire at baseline. Participants who at 3-YFU were still alive and living in Västerbotten (*n* = 3,181) were sent a follow-up questionnaire, of whom 2,336 (73.4%) responded. Those who at 6-year follow-up (6-YFU), in 2016, were still alive and lived in Västerbotten (*n* = 2,226) were sent a second follow-up questionnaire, of whom 1,837 (82.5%) responded. Table 1 describes this sample, which was used in the following analyses, in terms of numbers of respondents across age and sex strata at baseline, 3- and 6-YFU.

**Table 1:** Numbers of participants across age and sex strata at three time-points of assessment. The numbers in parentheses refer to percentage of those who agreed to participate among those invited in that age and sex strata at that time-point

	Baseline		Three-year follow-up		Six-year follow-up	
Age at baseline (years)	Women	Men	Women	Men	Women	Men
18–29	307 (32.1)	179 (17.3)	137 (59.5)	76 (54.7)	84 (70.0)	48 (72.7)
30–39	266 (40.3)	177 (24.7)	165 (66.3)	90 (53.9)	127 (78.4)	66 (73.3)
40–49	288 (40.5)	230 (31.0)	197 (71.9)	139 (61.8)	155 (80.3)	106 (76.8)
50–59	367 (50.9)	295 (39.5)	283 (79.3)	226 (77.4)	239 (86.6)	191 (86.4)
60–69	405 (58.4)	356 (50.7)	324 (82.2)	293 (84.2)	267 (84.8)	250 (89.0)
70–79	265 (53.8)	271 (63.9)	200 (50.8)	206 (80.8)	146 (81.1)	158 (85.9)
Total sample	1898 (45.2)	1508 (34.9)	1306 (74.5)	1030 (72.2)	1018 (81.7)	819 (83.6)

### Questions and questionnaire instrument

Single questions were used to assess demographics, physical exercise, self-rated health and diagnoses given by a physician of relevance for anxiety and depression. Physical exercise was assessed with the question “How often do you exercise?” (alternatives: Once a month or more seldom; 2–4 times/month; 2–3 times/week; More than 3 times/week), and self-rated health with the question “In general, how would you describe your health?” (alternatives: Poor; Fair; Good; Very good; Excellent). The diagnoses of generalized anxiety disorder, panic disorder, posttraumatic stress disorder, exhaustion disorder, and depression, were assed with the question “Have you been given the following diagnoses by a physician?” (alternatives: Yes; No).

The Swedish version [24] of the Hospital Anxiety and Depression Scale (HADS) [25] was used to assess symptoms of anxiety and depression. It consists of seven items for anxiety (HADS-A; e.g., “Worrying thoughts go through my mind”) and seven for depression (HADS-D e.g., “I have lost interest in my appearance”) regarding the past week. Each item is rated on a 4-point scale ranging from 0 to 3, with a total score for each subscale ranging from 0 to 21 (high score representing high level of anxiety and depression symptoms). A cut-off score of ≥ 8 was used for both subscales, providing an optimal balance between sensitivity and specificity for caseness, thus a high probability of being a case [26]. The HADS has good discriminant and concurrent validity and good internal consistency [26, 27]. Cronbach α for the present data at baseline was 0.84 for HADS-A and 0.83 for HADS-D.

### Procedure

An identical questionnaire was sent on paper by surface mail to the participants at each of the three assessments, and the filled-out questionnaire was returned in an envelope with prepaid postage. The survey included information about voluntary participation, confidentiality and intended use of data, as well as an informed consent statement. A reminder was sent to non-responders after three weeks, and an additional reminder and a new copy of the questionnaire after another three weeks. Data collection was conducted between March and April on all three occasions.

Missing values on the and HADS (1.33% at baseline, 1.26% at 3-YFU, and 1.92% at 6-YFU) were estimated with multiple imputations using fully conditional Markov chain Monte Carlo methods with 10 maximum iterations by means of which five imputed datasets were created [28]. The data was pooled across the five imputed datasets.

### Statistical analysis

Median and range scores on the HADS-A and HADS-D at baseline and 3- and 6-YFU for the entire sample were calculated to describe its general level of anxiety and depression symptoms. To optimize the reliability of the results on three-year associations with caseness, data from baseline to 3-YFU were combined with those from 3-YFU to 6-YFU. Prevalence rates for each of the four caseness conditions at baseline were then calculated for each conditions three years later, and overall differences between conditions were tested with chi-square analyses. This was followed-up by post-hoc, chi-square analyses to identify which of the four conditions after three years that differed significantly in their association with each of the conditions at baseline. Alpha-level was set at 0.05. To identify the most common six-year prognoses of caseness, prevalence rates at baseline, 3-YFU and 6-YFU were calculated for each of the 64 possible six-year prognoses. The study was not registered or protocol supported. The Statistical Package for the Social Sciences (IBM SPSS Statistics for Windows, Version 26, Armonk, NY) was used for the data analyses.

## Results

### Sample characteristics

The sample is described in Table 2 with respect to demographics, physical exercise, self-rated health, and diagnoses. Median (range) score on level of anxiety (HADS-A) / depression (HADS-D) symptoms were 3.0 (21) / 2.0 (20) at baseline, 3.0 (20) / 2.0 (19) at 3-YFU, and 3.0 (20) / 2.0 (18) at 6-YFU. At baseline, 9.53% (*n* = 175) of the participants met the criterion for anxiety caseness, 1.80% (*n* = 33) met the criterion for depression caseness, 5.33% (*n* = 98) met the criterion for comorbidity caseness, and 85.34% (*n* = 1531) met none of the criteria for anxiety or depression caseness. Corresponding prevalence rates were 9.53% (*n* = 175), 1.69% (*n* = 31), 5.77% (*n* = 106), and 80.02% (*n* = 1525) at 3-YFU, and 10.45% (*n* = 192), 2.61% (*n* = 48), 6.70% (*n* = 123), and 80.24% (*n* = 1,474) at 6-YFU, respectively.

**Table 2:** Characteristics of the total sample at baseline

Age, years, mean (SD)	54.8 (14.8)
Women, % (n)	1018 (55.4)
Married/living with partner, % (n)	1432 (78.0)
University education, % (n)	768 (41.8)
Physical exercise > 2 times/week, % (n)	1261 (68.6)
Self-rated health, % (n)	
Excellent/very good	755 (41.1)
Good	609 (33.2)
Fairly good/poor	454 (24.7)
Missing values	19 (1.0)
Self-report of physician-based diagnosis, % (n)	
Generalized anxiety disorder	13 (0.7)
Panic disorder	18 (1.0)
Posttraumatic stress disorder	14 (0.8)
Exhaustion disorder	80 (4.4)
Depression	72 (3.9)

### Three-year associations with caseness

Table 3 shows prevalence rates for each of the four caseness conditions associated with a certain caseness condition three years later, when combining data from baseline to 3-YFU with that from 3-YFU to 6-YFU. Chi-square analyses yielded significant differences across associated conditions for the anxiety ($\chi$ = 187.26, df = 3, *p* < 0.001), depression ($\chi$ = 41.50, df = 3, *p* < 0.001), comorbidity ($\chi$ = 55.22, df = 3, *p* < 0.001), and no caseness ($\chi$ = 6722.39, df = 3, *p* < 0.001).

**Table 3:** Proportion in percentage (n) of casenesses three years after a certain caseness. The proportions are based on data from baseline to three-year follow-up and from three-year follow-up to six-year follow-up. Results are shown from post-hoc chi-square analyses

Associated variable three years earlier	Three-year outcome				
	Anxiety	Depression	Comorbidity	None	Post-hoc $\chi^{2}$
Anxiety (A)	39.1 (137)	1.1 (4)	13.7 (48)	46.0 (161)	N = A > C > D
Depression (D)	9.4 (6)	18.7 (12)	12.5 (8)	59.4 (38)	N > A = C = D
Comorbidity (C)	21.1 (43)	5.4 (11)	36.8 (75)	36.8 (75)	N = C > A > D
None (N)	5.9 (181)	1.7 (52)	3.2 (98)	89.2 (2725)	N > A > C > D

Results from post-hoc, chi-square analyses are given in Table 3. Anxiety caseness was significantly more strongly associated with no caseness and with anxiety after three years than it was with comorbidity, which, in turn, was more strongly associated with anxiety than was depression. Depression was more strongly associated with no caseness than with depression, comorbidity and anxiety. Comorbidity was more strongly associated with no caseness and comorbidity than with anxiety, which, in turn, was more strongly associated with comorbidity than was depression. Finally, no caseness was more strongly associated with no caseness than with anxiety, which, in turn, was more strongly associated with no caseness than was comorbidity, and, in turn, more so than with depression.

### Most common six-year prognoses of caseness

Prevalence rates at baseline, 3-YFU and 6-YFU for each of the 64 possible six-year prognoses are shown in Table 4. To cover a relatively large proportion of all participants, *n* = 1,479 (80.5%), for good representativeness, yet in most cases to have large group sizes at each point in time, the two most common six-year prognoses for each caseness condition were identified. The two most common prognoses in each caseness condition at baseline are shown in Table 5, which also gives median scores on the HADS-A and HADS-D at each time point. The most common prognosis from baseline via 3-YFU to 6-YFU, irrespective of caseness or not, was to recover from being a caseness or remain not being a caseness at both 3-YFU and 6-YFU. The second most common prognosis in any caseness at baseline was to remain in that condition at both 3- and 6-YFU, and if not being a caseness at baseline to remain in that condition at 3-YFU and develop anxiety caseness at 6-YFU. Table 4 also shows that nine of the 64 six-year prognoses were not followed by any participant, 16 were followed by one participant, and five were followed by two participants.

**Table 4:** Prevalence rates for six-year prognosis in percentage (n) in relation to the entire sample (*n* = 1837) for anxiety, depression and comorbidity caseness, and no caseness at baseline, three-year follow-up (3-YFU) and six-year follow-up (6-YFU)

Anxiety caseness at baseline 9.53 (175)		Depression caseness at baseline 1.80 (33)		Comorbidity caseness at baseline 5.33 (98)		No caseness at baseline 83.34 (1531)	
3-YFU	6-YFU	3-YFU	6-YFU	3-YFU	6-YFU	3-YFU	6-YFU
Anxiety 3.59 (66)	Anxiety	Anxiety 0.16 (3)	Anxiety	Anxiety 1.03 (19)	Anxiety	Anxiety 4.74 (87)	Anxiety
	2.23 (41)		0.05 (1)		0.27 (5)		1.31 (24)
	Depression		Depression		Depression		Depression
	0 (0)		0 (0)		0.05 (1)		0.05 (1)
	Comorbidity		Comorbidity		Comorbidity		Comorbidity
	0.27 (5)		0.05 (1)		0.49 (9)		0.76 (14)
	No condition		No condition		No condition		No condition
	1.09 (20)		0.05 (1)		0.22 (4)		2.61 (48)
Depression 0.11 (2)	Anxiety	Depression 0.33 (6)	Anxiety	Depression 0.22 (4)	Anxiety	Depression 1.03 (19)	Anxiety
	0 (0)		0 (0)		0 (0)		0.16 (3)
	Depression		Depression		Depression		Depression
	0.05 (1)		0.16 (3)		0.05 (1)		0.05 (1)
	Comorbidity		Comorbidity		Comorbidity		Comorbidity
	0.05 (1)		0.05 (1)		0 (0)		0.05 (1)
	No condition		No condition		No condition		No condition
	0 (0)		0.11 (2)		0.16 (3)		0.76 (14)
Comorbidity 1.03 (19)	Anxiety	Comorbidity 0.27 (5)	Anxiety	Comorbidity 2.01 (37)	Anxiety	Comorbidity 2.45 (45)	Anxiety
	0.49 (9)		0 (0)		0.49 (9)		0.33 (6)
	Depression		Depression		Depression		Depression
	0.05 (1)		0.11 (2)		0.11 (2)		0.11 (2)
	Comorbidity		Comorbidity		Comorbidity		Comorbidity
	0.33 (6)		0.05 (1)		1.20 (22)		0.49 (9)
	No condition		No condition		No condition		No condition
	0.16 (3)		0.11 (2)		0.22 (4)		1.52 (28)
No condition 4.79 (88)	Anxiety	No condition 1.03 (19)	Anxiety	No condition 2.07 (38)	Anxiety	No condition 75.12 (1380)	Anxiety
	1.03 (19)		0.05 (1)		0.33 (6)		3.70 (68)
	Depression		Depression		Depression		Depression
	0.05 (1)		0.05 (1)		0.16 (3)		1.52 (28)
	Comorbidity		Comorbidity		Comorbidity		Comorbidity
	0.49 (9)		0 (0)		0.27 (5)		2.12 (39)
	No condition		No condition		No condition		No condition
	3.21 (59)		0.93 (17)		1.31 (24)		67.77 (1245)

**Table 5:** The first and second most common prognoses from baseline, via three-year follow-up to six-year follow-up as the endpoint, and median (range) scores on severity of anxiety and depression symptoms

Baseline	3-year follow-up	6-year follow-up
Anxiety caseness only		
First	No caseness	No caseness
A: 8 (4); D: 3 (7)	A: 5 (7); D: 2 (6)	A: 4 (7); D: 2 (6)
Second	Anxiety caseness	Anxiety caseness
A: 10 (7); D: 4 (6)	A: 10 (6); D: 4 (6)	A: 10 (8); D: 3 (7)
Depression caseness only		
First	No caseness	No caseness
A: 5 (6); D: 8 (5)	A: 2 (7); D: 3 (6)	A: 2 (7); D: 2 (7)
Second	Depression caseness	Depression caseness
A: 5 (3); D: 13 (6)	A: 3 (0); D: 11 (3)	A: 5 (1); D: 13 (7)
Comorbidity caseness		
First	No caseness	No caseness
A: 10 (9); D: 12 (8)	A: 3 (6); D: 2 (7)	A: 3 (7); D: 3 (7)
Second	Comorbidity caseness	Comorbidity caseness
A: 13 (12); D: 11 (12)	A: 15 (12); D: 10 (11)	A: 13 (10); D: 11 (8)
No caseness		
First	No caseness	No caseness
A: 2 (7); D: 1 (7)	A: 2 (7); D: 1 (7)	A: 2 (7); D: 1 (7)
Second	No caseness	Anxiety caseness
A: 5 (7); D: 2 (7)	A: 5 (7); D: 2 (7)	A: 8.5 (7); D: 4 (7)

A = Anxiety subscale of the Hospital Anxiety and Depression Scale

D = Depression subscale of the Hospital Anxiety and Depression Scale

## Discussion

To the best of our knowledge, this is the first population-based study of prognosis of anxiety and depression caseness and their comorbidity over this long a time-period, and assessed at more than two points in time. For the three-year associations, we took advantage of all available data, thus, from baseline to 3-YFU and from 3-YFU to 6-YFU. The results showed that when being an anxiety caseness, it was most common, and about equally common, to recover as it was to remain with anxiety. It was less common to develop comorbidity, and least common to recover from the anxiety and instead develop depression. Being a depression caseness most commonly resulted in recovery, and less common to either recover from depression and instead develop anxiety, develop also anxiety, or remain with depression. However, this calls for caution considering the small number of cases with depression at baseline (n = 33) and 3-YFU (n = 31). Being a comorbidity caseness, it was most common, and about equally common, to recover as to remain with comorbidity. It was less common to recover from depression, but remain with anxiety, and least common to recover from anxiety, but remain with depression. Being neither an anxiety nor depression caseness, it was most common to remain so after three years, less common to develop anxiety, even less common to develop comorbidity, and least common to develop depression.

Overall, the results from the three-year associations show that it was most common to recover when being an anxiety, depression or comorbidity caseness, and when not being a caseness it was most common to remain so. Apart from this, it was rather common to remain in the same caseness condition after three years, and this was particularly so for comorbidity caseness. Anxiety and depression caseness differed, such that in comorbidity it was more likely to recover from depression than from anxiety. In addition, being no caseness was more likely to develop into anxiety than into depression caseness.

Regarding the prognoses across six years, most of them were simply concordant with the three-year associations, whereas the most common additional pattern was for the affective caseness to continue from 3-YFU to 6-YFU. Notably, when being no caseness at baseline, the second most common prognosis, after continued no caseness at 3-YFU, was to develop anxiety at 6-YFU.

Some of the progressions were considerably more common than other progressions. Thus, close to half of the 64 possible six-year prognoses were followed by only two or fewer of the participants. In contrast, taken together, 80.5% of the participants followed either the most common or the second most common six-year prognosis for the four caseness conditions. This implies good representativeness in these prognoses for the general population.

Although different types of samples, designs and methods have been applied, the present results show similarities with prior work. Comorbidity between anxiety and depression has been reported to have a poorer recovery prognosis than depression alone [8, 12, 13], which the present recovery results for three-year associations support (36.8% for comorbidity vs 59.4% for depression). Furthermore, in accordance with earlier studies [29–31], relapse in depression was more common in comorbid anxiety (9 cases) than without comorbid anxiety (1 case). Global prevalence and incidence rates for depression are quite similar, whereas the prevalence rate for anxiety is much higher than the incidence rate, indicating that anxiety is a more persistent condition than depression [1]. This may partly be explained by anxiety being a trait [32], thereby easily evoked by environmental triggers due to dysregulated cognition and affect. There is also genetic and epidemiological evidence linking trait anxiety with stress-related vulnerability to depression [32].

The present results on recovery across three years are also in line with this difference (59.4% for depression vs 46.0% for anxiety). A meta-analysis also showed that anxiety predicts depression better than vice versa, although the effect size was very small and unlikely to be clinically meaningful [16]. The present data support this description based on the proportions of participants who develop comorbidity three years after anxiety compared to after depression (13.7% develop comorbidity after anxiety vs 12.5% after depression). However, the likelihood of developing depression and anxiety without comorbidity exhibits the opposite pattern, but the small sample size for depression (*n* = 64) calls for caution.

There are numerous theories regarding the very close relationship between anxiety and depression [9, 11]. For example, Gold [33] argues that depression and anxiety can cause a vicious circle due to increased activity in amygdala. According to other theories, avoidance behavior as a result of anxiety causes decreased positive affect, leading to depression. In the opposite direction, social withdrawal as a result of depression causes fear and anxiety linked to social situations [11]. In line with this, another Swedish population-based survey showed that about half of the participants who met the criterion for depression caseness (“clinically significant depression”) also met the criterion for anxiety caseness (“clinically significant anxiety”) [4], whereas the present study showed as many as three out of four participants fulfilling this description.

The present findings suggest that in the general adult population, about 54% of those with a high probability of being a clinical case with anxiety, about 40% of those with depression, and about 63% with comorbidity of anxiety and depression will not recover within a three-year period. The results further show that the second most common six-year prognoses in these subpopulations are to remain in these conditions also after six years. An implication of these results is need of further efforts in prevention of long-term affective health problems. Among those with anxiety and depression comorbidity at baseline, about 37% had this condition also after six years, and 22% in between these endpoints as well. This makes this comorbidity a particular public health problem since it is strongly associated with severe somatic symptomatology, very poor quality of life, and increased risk for suicide [3, 4, 7, 8, 14, 15].

The present study intended to describe relatively long time-period changes in common affective health conditions in a general adult population, in order to provide further understanding relevant for healthcare planning and disease prevention. Thus, the present focus was the associations between these conditions, not risk factors for the progression of each of them per se. Common ones being identified for affective conditions are treatment history, demographics (e.g., age, sex and marital status) and socioeconomics [29, 34, 35].

As part of guidelines for prognosis studies [36] the present study had a prospective design and used a large sample. The prospective design limits the risk of recall bias, and being population-based is a particular advantage regarding mental health issues, since individuals with psychiatric disorders are often reluctant to seek treatment, especially men and the elderly [4, 6]. The study also had a population-based sample stratified for age and sex, recruited from a population with an age and sex distribution that is very similar to that of Sweden in general [20].

A number of limitations with the present study should be considered. First, the relatively low response rate of 40.0% at baseline has implications for the representativeness due to potential selection bias, in particular among men aged 18–29 years, for whom the participation rate at baseline was 17.3% (although higher at 3-YFU, 54.7%, and 6-YFU, 72.7%). The response rate increased, however, to 73.4% at 3-YFU, and to 82.5% at 6-YFU for the total sample. This may be a concern from a clinical perspective, as young adults tend to respond poorer to psychological therapy than do older adults [37, 38]. Second, the relatively long time interval (three years) between assessments may be seen as a limitation, insomuch as several changes in the respondents’ conditions might have occurred in the interval. It should be noted that the items referred only to the past week. It is possible that the participant may have recovered from or developed anxiety and/or depression, resulting in an inaccurate prognosis. Future studies may use shorter time interval between assessments, and perhaps also assess the condition over a longer period than one week, as was done in the present study. Third, although statistical analyses were not conducted regarding the most common six-year prognoses of caseness, certain caution should be taken when interpreting the results due to the relatively small sample sizes for depression only (*n* = 33) and comorbidity (*n* = 98). Fourth, and finally, the use of questionnaire instruments to assess anxiety and depression brings both advantages and disadvantages. As much as they are validated, they inform on high probability of being a clinical case, even if the respondent has not actually received a diagnosis from a physician. This reduces under-reporting due to not (yet) having sought help or other administrative lacunae related to health journal reporting and communication. On the other hand, it is open to erroneous self-report, rather than explicitly meeting diagnostic criteria based on a diagnostic interview. However, such error is arguably unlikely to be systematic in this kind of population survey that is independent from clinical practice.

## Conclusion

Despite certain limitations, the results of the study suggest that in the general Swedish adult population, only about 46%, 60% and 37% with high probability of being a clinical case with anxiety, depression, and their comorbidity, respectively, will recover within a three-year period. In addition, common prognoses implicate remaining in these conditions of ill-health after six years. The poor prognosis of comorbidity in combination with its very poor quality of life identifies comorbid anxiety and depression as a particular public health problem.

## Data Availability

The dataset used and/or analyzed during the current study are available from the corresponding author on reasonable request.

## Abbreviations

HADS: Hospital Anxiety and Depression Scale
HADS-A: Anxiety Subscale of the Hospital Anxiety and Depression Scale
HADS-D: Depression Subscale of the Hospital Anxiety and Depression Scale
YFU: Year Follow-Up

## References

[1] Disease and Injury Incidence and Prevalence Collaborators (2017). Global, regional, and national incidence, prevalence, and years lived with disability for 354 diseases and injuries for 195 countries and territories, 1990–2017: a systematic analysis for the Global Burden of Disease Study 2017. Lancet.

[2] Steel Z, Marnane C, Iranpour C, Chey T, Jackson JW, Patel V, Silove D (2014). The global prevalence of common mental disorders: a systematic review and meta-analysis 1980–2013. Int J Epidemiol.

[3] Kessler RC, Sampson NA, Berglund P, Gruber MJ, Al-Hamzawi A, Andrade L (2015). Anxious and non-anxious major depressive disorder in the World Health Organization World Mental Health Surveys. Epidemiol Psychiat Sci.

[4] Johansson R, Carlbring P, Heedman Å, Paxling B, Andersson G (2013). Depression, anxiety and their comorbidity in the Swedish general population: point prevalence and the effect on health-related quality of life. PeerJ.

[5] Vos T, Flaxman AD, Naghavi M, Lozano R, Michaud C, Ezzati M (2012). Years lived with disability (YLDs) for 1160 sequelae of 289 diseases and injuries 1990–2010: a systematic analysis for the Global Burden of Disease Study 2010. Lancet.

[6] Wang PS, Aguilar-Gaxiola S, Alonso J, Angermeyer MC, Borges G, Bromet EJ (2007). Use of mental health services for anxiety, mood, and substance disorders in 17 countries in the WHO world mental health surveys. Lancet.

[7] Ionescu DF, Niciu MJ, Henter ED, Zarate CA (2013). Defining anxious depression: a review of the literature. CNS Spectr.

[8] Gaspersz R, Nawijn L, Lamers F, Penninx BW (2018). Patients with anxious depression: overview of prevalence, pathophysiology and impact on course and treatment outcome. Cur Opinion Psychiatr.

[9] Cummings CM, Caporino NE, Kendall PC (2014). Comorbidity of anxiety and depression in children and adolescents: 20 years after. Psychol Bull.

[10] Tyrer P (2001). The case for cothymia: mixed anxiety and depression as a single diagnosis. Brit J Psychiatr.

[11] Demyttenaere K, Heirman E (2020). The blurred line between anxiety and depression: hesitations on comorbidity, thresholds and hierarchy. Int Rev Psychiatr.

[12] American Psychiatric Association (2013). Diagnostic and statistical manual of mental disorders.

[13] Ionescu DF, Niciu MJ, Mathews DC, Richards EM, Zarate CA (2013). Neurobiology of anxious depression: a review. Depression Anxiety.

[14] Zimmerman M, Martin J, McGonigal P, Harris L, Kerr S, Balling C (2019). Validity of the DSM-5 anxious distress specifier for major depressive disorder. Depression Anxiety.

[15] Haug TT, Mykletun A, Dahl AA (2004). The association between anxiety, depression, and somatic symptoms in a large population: the HUNT-II study. Psychosom Med.

[16] Jacobson NC, Newman MG (2017). Anxiety and depression as bidirectional risk factors for one another: a meta-analysis of longitudinal studies. Psychol Bull.

[17] Saha S, Lim CC, Cannon DL, Burton L, Bremner M, Cosgrove P (2021). Co-morbidity between mood and anxiety disorders: a systematic review and meta-analysis. Depress Anxiety.

[18] Williams P, Tarnopolsky A, Hand D (1980). Case definition and case identification in psychiatric epidemiology: review and assessment. Psychol Med.

[19] Palmquist E, Claeson AS, Neely G, Stenberg B, Nordin S (2014). Overlap in prevalence between various types of environmental intolerance. Int J Hyg Environ Health.

[20] Statistics Sweden. Tables on the population of Sweden 2009: 1.3.1 Population by sex, age, marital status by county Dec. 31, 2009 according to the administrative subdivisions of Jan 1, 2010. Statistics Sweden, Örebro. https://www.scb.se/en/.

[21] Hillert L, Berglind N, Arnetz BB, Bellander T (2002). Prevalence of self-reported hypersensitivity to electric or magnetic fields in a population-based questionnaire survey. Scand J Work Environ Health.

[22] Naing L, Winn T, Rusli BN (2006). Practical issues in calculating the sample size for prevalence studies. Arch Orofacial Sci.

[23] Daniel WW (1999). Biostatistics: a foundation for analysis in the health sciences.

[24] Lisspers J, Nygren A, Soderman E (1997). Hospital Anxiety and Depression Scale (HAD): some psychometric data for a Swedish sample. Acta Psychiatr Scand.

[25] Zigmond AS, Snaith RP (1983). The Hospital Anxiety and Depression Scale. Acta Psychiatr Scand.

[26] Bjelland I, Dahl AA, Haug TT, Neckelmann D (2002). The validity of the Hospital Anxiety and Depression Scale: an updated literature review. J Psychosom Res.

[27] Hansson M, Chotai J, Nordstöm A, Bodlund O (2009). Comparison of two self-rating scales to detect depression: HADS and PHQ-9. Brit J Gen Pract.

[28] Schunk D (2008). A Markov chain Monte Carlo algorithm for multiple imputation in large surveys. AStA Adv Stat Analys.

[29] Buckman JEJ, Underwood A, Clarke K, Saunders R, Hollon SD, Fearon P (2018). Risk factors for relapse and recurrence of depression in adults and how they operate: a four-phase systematic review and meta-synthesis. Clin Psychol Rev.

[30] Moriarty AS, Meader N, Snell KI, Riley RD, Paton LW, Chew-Graham CA (2021). Prognostic models for predicting relapse or recurrence of major depressive disorder in adults. Cochrane Database Syst Rev.

[31] Saunders R, Cohen ZD, Ambler G, DeRubeis RJ, Wiles N, Kessler D (2021). A patient stratification approach to identifying the likelihood of continued chronic depression and relapse following treatment for depression. J Pers Med.

[32] Weger M, Sandi C (2018). High anxiety trait: a vulnerable phenotype for stress-induced depression. Neurosci Biobehav Rev.

[33] Gold P (2015). The organization of the stress system and its dysregulation in depressive illness. Mol Psychiatr.

[34] Buckman JEJ, Saunders R, Stott J, Arundell L-L, O’Driscoll C, Davies MR (2021). Role of age, gender and marital status in prognosis for adults with depression: An individual patient data meta-analysis. Epidemiol Psychiatr Sci.

[35] Buckman JEJ, Saunders R, Stott J, Cohen ZD, Arundell L-L, Eley TC (2022). Socioeconomic indicators of treatment prognosis for adults with depression. JAMA Psychiat.

[36] Riley RD, Hayden JA, Steyerberg EW, Moons KG, Abrams K, Kyzas PA (2013). Prognosis Research Strategy (PROGRESS) 2: prognostic factor research. PLoS Med.

[37] Buckman JEJ, Stott J, Main N, Antonie DM, Singh S, Naqvi SA, et al. Understanding the psychological therapy treatment outcomes for young adults who are not in education, employment, or training (NEET), moderators of outcomes, and what might be done to improve them. Psychol Med. 2021;1–12. 10.1017/S0033291721004773. 10.1017/S0033291721004773 PMC10235648 37449486

[38] Saunders R, Buckman JEJ, Stott J, Leibowitz J, Aguirre E, John A (2021). Older adults respond better to psychological therapy than working-age adults: evidence from a large sample of mental health service attendees. J Affect Disord.

